# Real-world evaluation of select adverse drug reactions and healthcare utilization associated with parenteral Ibuprofen and ketorolac in adult and pediatric patients

**DOI:** 10.3389/fpain.2024.1484948

**Published:** 2025-01-07

**Authors:** Mosadoluwa Afolabi, Jensy Rodriguez-Silva, Ishveen Chopra, Ines Macias-Perez, Jason Makii, Emily Durr, Theresa Human

**Affiliations:** ^1^ STATinMED, LLC, Dallas, TX, United States; ^2^ Cumberland Pharmaceuticals, Nashville, TN, United States

**Keywords:** NSAID, adverse drug reaction (ADR), intravenous ibuprofen, ketorolac, side effect, healthcare resource utilization, patient safety

## Abstract

**Introduction:**

Intravenous non-steroidal anti-inflammatory drugs (NSAIDs) are commonly used in healthcare settings, but their comparative safety and resource utilization impacts remain understudied. This study aimed to compare adverse drug reactions (ADRs) and healthcare resource utilization (HCRU) between patients receiving IV-ibuprofen versus IV/IM ketorolac.

**Methods:**

A retrospective, longitudinal analysis was conducted using an all-payer database, examining records from January 1, 2014, to June 3, 2023. The study included both adult (≥18 years) and pediatric (<18 years) populations who received one or more doses of either medication. Propensity score matching was applied to both populations, and HCRU was tracked for 29 days post-final dose. The adult cohort included 31,046 IV-ibuprofen and 124,184 ketorolac records, while the pediatric cohort had 5,579 patients per treatment arm.

**Results:**

Both adult and pediatric patients receiving IV-ibuprofen demonstrated lower ADR incidence and reduced HCRU compared to those receiving ketorolac.

**Discussion:**

The findings suggest IV-ibuprofen may be a safer alternative to ketorolac, potentially improving patient care outcomes while reducing healthcare system burden. These results have implications for clinical practice and healthcare resource management.

## Background

Adverse drug reactions (ADRs) are unintended injuries or complications that arise from administration of a medication. These unwanted drug effects have considerable clinical costs as they can lead to emergency department visits, hospital admission, prolongation of hospital stays and can result in disability or even death (1–4). A recent study estimated that the annual impact of serious ADRs, among hospitalized adult and pediatric patients in the United States (US), results in 2.2 million hospitalizations and 106,000 deaths (5). The economic burden of ADRs is also substantial, with estimates of up to $30.1 billion annually in the US alone (6). Additionally, ADRs can have a significant effect on a patients’ quality of life, including physical and emotional suffering, social isolation, and impaired daily functioning (7). Choosing the most appropriate medication is of the utmost importance to minimize harm, improve outcomes, and reduce medical costs and resources.

Currently there are two intravenous (IV) nonsteroidal anti-inflammatory drugs (NSAID) on the US market, ketorolac injection and IV ibuprofen (Caldolor®, Cumberland Pharmaceuticals Inc., Nashville, TN, USA). Only the parental formulation of ketorolac will be considered in this study. Ketorolac was approved in 1989 for the short-term management of moderately severe, acute pain requiring opioid analgesia (8). Although ketorolac is the most frequently administered IV NSAID, it has several limitations including multiple contraindications. Ketorolac is contraindicated for: use in excess of 5 days due to increased potential for frequency and severity of ADRs; use in pediatric patients; administration preoperatively due to increased risk of bleeding; use in patients with active or history of gastrointestinal (GI) bleeding or peptic ulcer disease; use in patients at high risk of bleeding and those with advanced renal impairment; and use in labor and delivery due to adverse effects on fetal circulation, inhibition of uterine contractions, and increased risk of uterine hemorrhage (8–11). Intravenous (IV) ibuprofen was first approved in 2009 for the management of mild to moderate pain, moderate to severe pain in combination with narcotics, and for the reduction of fever in adults and pediatric patients ≥3 months of age (12, 13). Additionally, IV ibuprofen has demonstrated efficacy and safety when administered pre-surgically (14, 15).

The mechanism of action of non-selective NSAIDs include inhibition of cyclooxygenase 1 (COX-1) and COX-2 pathways which decrease the expression of prostaglandin precursors, including thromboxane and prostacyclin, and is how they derive their analgesic, antipyretic, and anti-inflammatory properties (16). While all IV NSAIDs are non-selective in their COX-1 and COX-2 inhibition, they differ in their degree of inhibition of COX-1 relative to COX-2. Warner et al. evaluated the COX isoenzyme inhibitory capacity of multiple NSAIDs and reported ketorolac as the most COX-1 selective, which aligns with studies that signal ketorolac to be the most gastro- and nephrotoxic of all NSAIDs (9, 17, 18). Ibuprofen inhibits COX-1 2.5 times more than COX-2 and would be considered slightly more neutral than the other NSAIDs (19). A meta-analysis of 28 studies evaluating the relative risk (RR) of GI adverse reactions in several NSAIDs, demonstrated ibuprofen had one of the most favorable risk profiles (RR 1.84; 95% CI 1.54, 2.20) and significantly safer than ketorolac (RR 11.50; 95% CI 5.56, 23.78) (20).

Although these medications have been extensively studied, there are no studies that describe the difference in adverse reactions between agents. The objective of this study is to describe and compare ADRs after exposure to IV ibuprofen vs. IV or intramuscular (IM) ketorolac, in both adult and pediatric populations. The economic impact of the difference will also be described through all-cause and ADR-related health care resource utilization (HCRU) within 29 days after the last dose of medication administration.

## Methods

### Study design and data source

This is a retrospective, longitudinal, observational database study evaluating an all-payer database assessing patients that received one or more doses of IV ibuprofen or IV/IM ketorolac between January 1, 2014 to June 30, 2023.

Real World Data (RWD) Insights data was utilized for this study. RWD Insights includes comprehensive coverage at the patient level across the US health care system, as well as all provider types. Data are de-identified and complies with the requirements of the Health Insurance Portability and Accountability Act (HIPAA).

### Study sample

**Figure 1:**
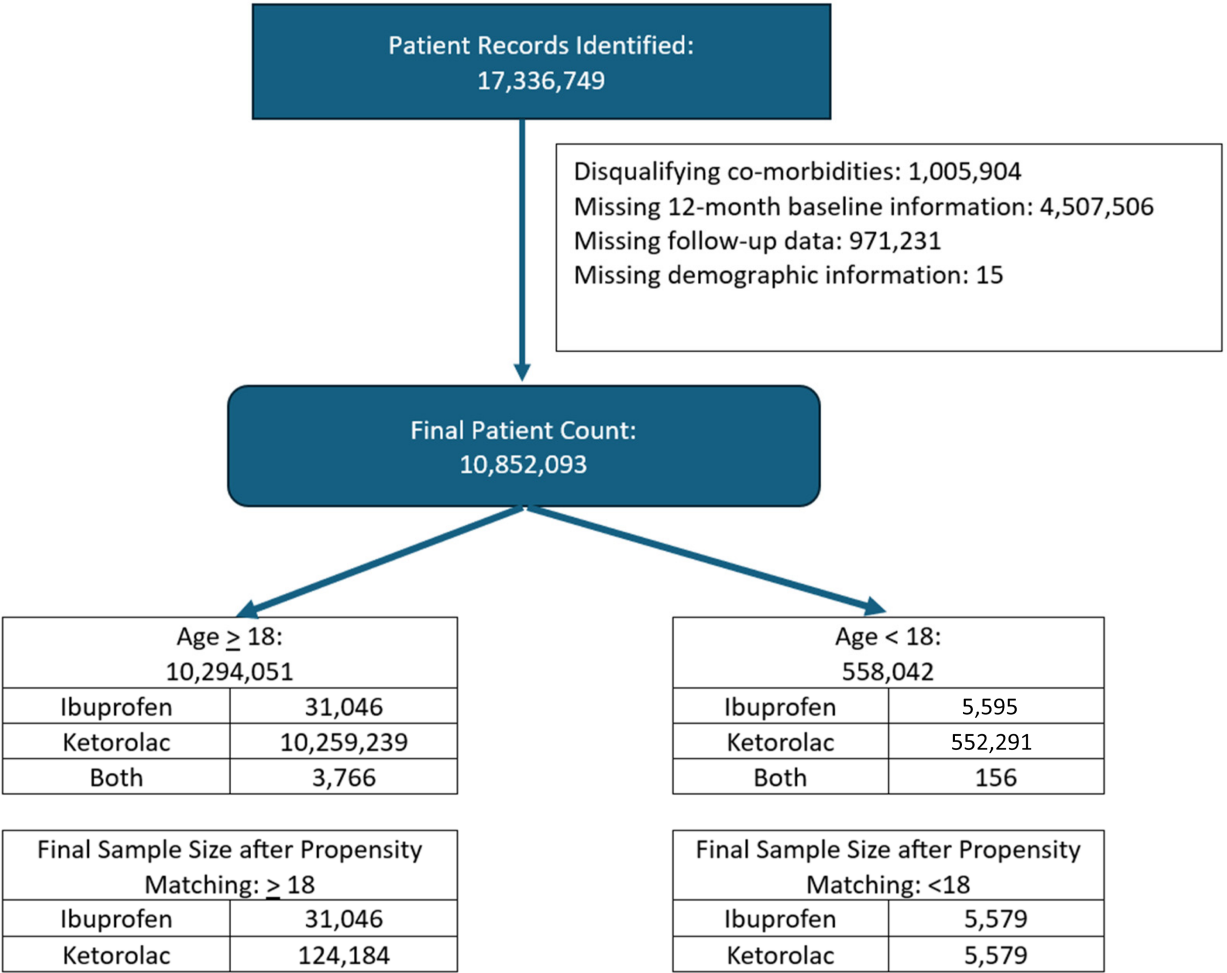
Patient selection.

Eligible patients were classified into 2 age groups: <18 years and ≥18 years old. Patients had at least ≥1 pharmacy or medical claim for IV ibuprofen or IV/IM ketorolac and ≥12-months pre/post-index date administration data records. Patients were excluded if they had a pre-index ICD-9/ICD-10 claim for renal dysfunction, GI or general bleeding disorders, low back pain, headache, abdominal pain, nausea/vomiting, or throat pain. Patients that received oral/ophthalmic/nasal formulations or received both medications were also excluded. The first dose administered is considered the index dose (Figure 1).

### Study measures

Key baseline patient characteristics included demographics, dose, number of doses administered, diagnoses (ICD-9/ICD-10 codes), physician characteristics, setting of drug administration, reason for drug administration (surgical, fever, non-surgical), payer channel, concurrent medication use (ACEI, ARB, calcineurin inhibitors, antiplatelet, anticoagulants, NSAIDs, IIb/IIIa inhibitors, aminoglycoside, vancomycin, acyclovir), Charlson Comorbidity Index (CCI) Score, CCI comorbidities, and Pediatric Comorbidity Index (PCI) (age <18 years).

Safety with these medications was assessed during the post-index period. Common ADRs for both parenteral ibuprofen and ketorolac were identified *a priori* and included renal dysfunction, GI bleeding disorders, general bleeding disorders, low back pain, headache, abdominal pain, nausea/vomiting, and throat pain. These ADRs were identified using ICD-9/ICD-10 codes. The incidence of ADRs post parenteral ibuprofen and ketorolac dose was identified 72 h after the last dose administered (maximum of 5 days). As previously described, patients with a claim for any of the targeted side effects 12-month prior to the index dose were excluded.

Healthcare resource utilization (HCRU) were evaluated out to 29 days after last dose of treatment. All-cause HCRU during the follow-up period across all healthcare settings [hospitalizations/inpatient visits, outpatient visits, emergency department (ED) visits, and pharmacy] was assessed. All-cause visits were defined as any inpatient or outpatient visit related to any diagnosis.

### Statistical analyses

Descriptive analysis was performed on both demographic and outcome variables. Propensity score matching (PSM) was employed to account for observable baseline differences between the two groups of interest, with a matching ratio of 1:4 (ibuprofen: ketorolac) for adult patients and 1:1 (ibuprofen: ketorolac) for pediatric patients. The variables used in the logistic regression model included age group, sex, reason for administration (categorized as surgical, fever, or other), and the number of index drug administrations (ranging from 1 to 5+).

## Results

A total of 31,046 IV Ibuprofen and 124,184 parenteral ketorolac adult patients were identified and matched (1:4). In adults the mean dose was 784 ± 445 mg and 30 ± 16 mg for parenteral ibuprofen and ketorolac, respectively. One dose was administered to 93% of adult patients. Among adults, most patients were female, greater than 65 years of age, received the index drug for surgical pain, and had few to no comorbidities (Table 1, Supplementary Table S1). A significant portion of adults that received ketorolac had Medicare or Medicaid.

**Table 1:** Demographics adult and pediatric after matching.

	Adult	Adult	Pediatric	Pediatric
	IV ibuprofen	Ketorolac	IV ibuprofen	Ketorolac
	*N* = 31,046	*N* = 124,184	*N* = 5,579	*N* = 5,579
	*N* (%)	*N* (%)	*N* (%)	*N* (%)
Mean age	52 ± 16	52 ± 16	8 ± 5	8 ± 5
Race				
White	19,768 (84%)	80,384 (87%)	278 (88%)	259 (88%)
Black	3,229 (14%)	10,314 (11%)	33 (10%)	32 (10%)
Asian	633 (3%)	1,936 (2%)	4 (1%)	3 (1%)
Median CCI	0	0	0	0
Payer Channel				
Medicaid	5,957 (19%)	29,751 (24%)	3,414 (61%)	3,293 (59%)
Medicare	6,033 (19%)	28,963 (23%)	110 (2%)	343 (6%)
Private Insurance	17,456 (56%)	60,695 (49%)	1,874 (34%)	2,027 (36%)

In the pediatric population, 5,579 patients were identified in the IV ibuprofen group and matched (1:1). The mean dose was 429 ± 340 mg and 25 ± 12 mg for parenteral ibuprofen and ketorolac, respectively in the pediatric population. One dose was administered to 98% of pediatric patients. The pediatric population was slightly more male than females, aged 0–5 years old, received the index drug primarily for pain (both surgical and non-surgical), and had few to no comorbidities (Table 1, Supplementary Table S1).

## ADR differences

### Incidence of ADRs in adult population

Within three days of the last dose administered, claims for renal dysfunction events were reduced by 45% (0.34% vs. 0.62%; *p* < 0.001) in patients that received IV ibuprofen compared to IV/IM ketorolac. GI bleeding events were numerically lower in patients that received IV ibuprofen compared to ketorolac, although no difference statistically. Patients that received IV ibuprofen had a 78% reduction in hematuria (0.28% vs. 1.24%; *p* < 0.001), 77% reduction in abdominal/pelvic pain claims (2.6% vs. 11%; *p* < 0.001), 90% reduction in headache claims (0.38% vs. 4%; *p* < 0.001) and 80% reduction in nausea and vomiting (0.98% vs. 4.9%; *p* < 0.001) compared to ketorolac (Table 2).

**Table 2:** Adult incidence of common NSAID ADRs comparing IV ibuprofen to ketorolac.

Selected condition	IV ibuprofen event/denominator (%)	Ketorolac event/denominator (%)	% diff	*p*-value
Renal dysfunction	107/31,046 (0.3%)	776/124,184 (0.6%)	45%	<0.001
GI bleeding	79/31,046 (0.3%)	346/124,184 (0.3%)	NS	0.504
Hematuria	83/29,867 (0.3%)	1,470/118,711 (1.2%)	77%	<0.001
Low back pain	100/24,073 (0.4%)	2,653/91,338 (2.9%)	86%	<0.001
Pelvic/abdominal pain	504/19,596 (2.6%)	8,624/78,138 (11%)	77%	<0.001
Headache	96/25,170 (0.4%)	3,768/95,688 (3.9%)	90%	<0.001
Nausea/vomiting	240/24,527 (1%)	4,594/93,761 (4.9%)	80%	<0.001

### Incidence of ADRs in pediatric population

There were no differences in gastrointestinal bleeding, renal toxicity or lower back pain events. Claims for hematuria were lower by 64% (0.16% vs. 0.47%; *p* < 0.001). However, claims for abdominal/pelvic pain were lower by 67% (3.5% vs. 10%; *p* < 0.001), headache by 61% (2.2% vs. 5.7%; *p* < 0.001), and nausea/vomiting by 51% (4.6% vs. 9.4%; *p* < 0.001) in patients that received IV ibuprofen compared to IV/IM ketorolac (Table 3).

**Table 3:** Pediatric incidence of common NSAID ADRs comparing IV ibuprofen to ketorolac.

Selected condition	IV ibuprofen event/denominator	Ketorolac event/denominator	% diff	*p*-value
Renal dysfunction	2/5,579 (0.04%)	4/5,579 (0.07%)	NS	0.683
GI bleeding	5/5,579 (0.09%)	8/5,579 (0.14%)	NS	0.579
Hematuria	9/5,542 (0.16%)	26/5,526 (0.47%)	65%	0.007
Low back pain	18/3,339 (0.54%)	21/3,489 (0.60%)	NS	0.854
Pelvic/abdominal pain	98/2,804 (3.50%)	286/2,740 (10.44%)	66%	<0.001
Headache	69/3,099 (2.23%)	176/3,114 (5.65%)	60%	<0.001
Nausea/vomiting	126/2,746 (4.59%)	255/2,720 (9.38%)	51%	<0.001

### Healthcare resource utilization (HCRU) in adult population

In adult patients, the total HCRU within 29 days following the last dose of drug administration were significantly lower overall in the group that received IV ibuprofen when compared to those that received ketorolac. Length of stay (LOS) decreased by approximately one day (3.1 ± 4.8 vs. 3.8 ± 5.37; *p* < 0.001) in the IV Ibuprofen arm compared to ketorolac. Hospital visits (81% vs. 83%; *p* < 0.001), emergency room (ER) visits (7.5% vs. 25%; *p* < 0.001), and outpatient office visits (29% vs. 38%; *p* < 0.001) were significantly different among patients that received IV ibuprofen compared to ketorolac. Any inpatient admission however was slightly higher in the IV ibuprofen arm compared to ketorolac (9.1% vs. 8.5%; *p* = 0.001). There were no significant differences between the two groups in claims for hemodialysis, endoscopy, or transfusion of red blood cells in the 29 day follow up. (Table 4)

**Table 4:** HCRU in adult and pediatric patients.

	Adult	Adult	*P*-value	Pediatric	Pediatric	*p*-value
	IV Ibuprofen	Ketorolac		IV ibuprofen	Ketorolac	
	*N* = 31,046	*N* = 124,184		*N* = 5,579	*N* = 5,579	
	*N* (%)	*N* (%)		*N* (%)	*N* (%)	
Inpatient admission	2,811 (9.1%)	10,498 (8.5%)	0.001	258 (4.6%)	422 (7.6%)	<0.001
Hospital visit	25,070 (80.8%)	102,762 (82.8%)	<0.001	4,375 (78%)	5,031 (90%)	<0.001
ER visit	2,321 (7.5%)	31,408 (25.3%)	<0.001	1,724 (31%)	1,813 (33%)	0.073
Outpatient office visit	8,955 (28.8%)	47,639 (38.4%)	<0.001	1,759 (32%)	1,732 (31%)	0.596
Dialysis claims	1 (0.0%)	9 (0.01%)	0.693	0	0	NA
EGD/colonoscopy	380 (1.2%)	1,633 (1.3%)	0.215	9 (0.16%)	23 (0.41%)	0.021
Receive RBC	60 (0.19%)	238 (0.19%)	1	4 (0.07%)	8 (0.14%)	0.386

### Healthcare resource utilization in pediatric population

Total HCRU was lower in the IV-ibuprofen arm compared to the ketorolac group among the pediatric patients and was demonstrated by decreased LOS (2.1 ± 2.6 vs. 3.3 ± 4.4;<0.001), fewer hospital visits (78% vs. 90%; *p* < 0.001) and fewer ER visits (31% vs. 33%; *p* = 0.005). Significantly more claims for EGDs/colonoscopies were found among those that received ketorolac compared to IV ibuprofen (Table 4).

## Discussion

This retrospective, observational, payer-based database study shows a reduction in major ADRs in both the adult and pediatric groups. Among the adult population, nephrotoxicity was meaningfully reduced in patients that received IV ibuprofen compared to ketorolac, when undergoing a surgical procedure. Other common ADRs that are associated with NSAIDs were also significantly less reported. Reducing ADRs encountered by patients contributes substantially to patient satisfaction, recovery time, and minimizes healthcare resources (1–4).

As described previously, the mechanism of action of non-selective NSAIDs is inhibition of COX-1 and COX-2 pathways which decreases the expression of prostaglandin precursors, including thromboxane and prostacyclin, producing anti-inflammatory and analgesia properties. COX-1 acts primarily in the control of renal glomerular filtration rate (GFR) while COX-2 plays a role in sodium and water excretion. Prostaglandins, particularly PGE2 and PGD2, act as vasodilators in the afferent arterioles, increasing renal perfusion. By inhibiting this pathway, NSAIDs can result in acute vasoconstriction, reduce GFR, and ultimately lead to acute renal injury (21). Risk factors for acute kidney injury associated with NSAIDs include advanced age, systemic arterial hypertension, comorbidities that reduce renal profusion, and dehydration. Although it is anticipated that all NSAIDs carry some risk for acute renal dysfunction, short-term (<5 days) administration of ketorolac has been reported to carry an incidence of 1.1% and longer durations and higher doses at 2.1% (22). Ketorolac also has the highest number of reported cases in the literature and are likely secondary to greater COX-1 inhibition than all other NSAIDs (18, 23–29). It was withdrawn from the market in many countries following association with hemorrhage and renal failure (30). The reported incidence in this study, although lower than previously reported, are still statistically different, favoring fewer renal events with IV ibuprofen than ketorolac. Additionally, although the incidence appears nominal, the costs associated are expected significant. Lastly, there were no differences in claims for hemodialysis suggesting that either the renal dysfunction is reversible or not severe enough to require dialysis.

GI bleeding is one of the adverse effects reported among NSAIDs. Similarly, to renal adverse events, the etiology is secondary to inhibition of the COX isoenzymes, particularly COX-1 inhibition. Gastrointestinal injury ranges from dyspepsia to fatal GI bleeding and perforation. Risk factors for GI bleeding due to NSAIDs include age ≥65, history of previous GI bleed or peptic ulcer disease, concomitant use of aspirin, anticoagulants or corticosteroids, and elevated NSAID dose (31). There is debate as to whether individual NSAIDs carry a greater risk. Ketorolac has attracted attention lately with reports of increased incidence of GI bleeding. Rodrigez et al. compared the relative risk of GI bleeding among individual agents and reported a RR 24.7(9.6–63.5) with ketorolac compared to RR 2.1 (0.6–7.1) with ibuprofen (17). When evaluating doses, ketorolac doses ≤20 mg revealed RR 20.0 (4.3–93.6) and doses >20 mg RR 28.1 (8.7–90.9). Ibuprofen was not considered to carry a statistically significant risk, therefore individual doses were not assessed. In our study however, there was a numerically higher risk of GI bleeding with ketorolac compared to IV ibuprofen in both populations, though not statistically nor probably clinically significant. There were however a statistically higher rate of EGDs/colonoscopy claims in pediatric patients that received ketorolac. Potential reasons may be that clinicians are identifying and monitoring for these ADRs when NSAIDs are administrered, particularly when prescribing ketorolac. Additionally, most pharmacies have employed dose guidance for ketorolac due to the numerous warnings and contraindications, including dose, duration of therapy based on age, renal function. Lastly, clinicians may not be informed about the contraindication of ketorolac in the pediatric population and perhaps utilization in this population is resulting in more GI follow up.

As gastrointestinal toxicity and nephrotoxicity are the most feared ADRs, other side effects must be considered. Both ketorolac and IV ibuprofen demonstrate similar ADRs in >1% of patients (excluding those related to GI bleeding and renal toxicity) including abdominal pain, nausea, vomiting, headache, and prolonged bleeding (8, 12). Therefore each of these were identified through extraction of ICD-9/ICD-10 codes in the post-dose 72-hour timeframe. As prolonged bleeding is not a straightforward payer code, hematuria was identified as a surrogate marker for minor bleeding. Results demonstrated all ADRs identified *a priori* were statistically reduced in adult patients and in four of the five categories in pediatrics, when IV ibuprofen was compared to ketorolac. It is unclear if these results are based on the direct comparison of ADRs between agents, or due to secondary benefits. For example, if IV ibuprofen reduced opioids in the post-surgical phase vs. ketorolac, this could account for the reduction in nausea, vomiting, and abdominal pain.

Multiple doses, particularly with ketorolac, have been linked with worse side effects. However, given that most patients had 1 dose, we believe dose may not impact ADRs in this study. This is something that can by further explored in futures studies. Prevention of in ADRs is not only important to minimize harm, improve patient satisfaction, and improve outcomes but also to reduce the need for healthcare resources. The resource utilization comparison revealed shorter LOS, reduced outpatient follow up, and fewer ED visits in IV Ibuprofen arm compared to ketorolac.

## Limitations

Specific costs could not be evaluated accurately due to the disparity in payer types. It is well known that negotiated payments are significantly lower in patients with government backed insurers compared to private providers (32). As a significantly higher proportion of adult patients that received ketorolac had Medicare or Medicaid, and more that received IV Ibuprofen had private commercial plans, the costs could not be accurately calculated.

Surgical bleeding is another concern when utilizing NSAIDs, particularly when administered pre- or intra-operatively. Both ketorolac and IV ibuprofen appear to be safe when administered post-operatively (14, 33–41). Only IV ibuprofen has demonstrated safety when administered pre-operatively (14, 15, 42–44). Pre-emptive ketorolac resulted in increased bleeding post-operatively and therefore is contraindicated to be administered in this manner (8, 45). The majority of the patients identified in this cohort were administered either ketorolac or IV ibuprofen for surgical pain. Post-surgical bleeding would naturally be a safety end point to evaluate; however, collecting payer data that could determine if the agent was given pre or post-procedure proved to be not possible. Also, determining the incidence of post-operative bleeding is not based on predetermined or clear ICD-9/ICP-10 coding. If there were a difference in post-operative bleeding among groups, they did not require a difference in transfusions of RBCs, indicating either bleeding was not different and/or was not serious or life-threatening.

Certain limitations are associated with claims data use. The presence of a diagnosis code on a medical claim is not a positive presence of disease, as the diagnosis code may be incorrectly coded or included as rule-out criteria rather than the actual disease. Additionally, administrative claims data do not contain clinical variables, such as weight, which would have been valuable in the pediatric population. Lastly, as back pain has been identified in both IV ibuprofen and ketorolac as a common side effect, it cannot be ruled out that these claims were coded as ADR rather than reason for drug administration.

## Conclusion

This real-world evidence study is the first to compare IV ibuprofen and ketorolac using an all-payor database which demonstrated that IV ibuprofen reduced the incidence of unfavorable outcomes and was associated with less healthcare utilization. Results revealed a significant reduction in renal dysfunction, hematuria, abdominal/pelvic pain, headache, nausea/vomiting, LOS, and outpatient costs in the adult population that received IV ibuprofen compared to ketorolac. Inpatient visits were higher however in the IV ibuprofen arm. The pediatric population also showed a reduction in abdominal/pelvic pain, headache, nausea/vomiting, LOS and overall HCRU, including reduction in EGD/colonoscopies in the 29-day follow up timeframe. In summary, the results of this study highlight the potential for IV ibuprofen to offer a safer compared to ketorolac, while reducing the burden on healthcare systems.

## Data Availability

The raw data supporting the conclusions of this article will be made available by the authors, without undue reservation.
