# Supplementary material for: Real-world evaluation of select adverse drug reactions and healthcare utilization associated with parenteral Ibuprofen and ketorolac in adult and pediatric patients
Source: Front Pain Res (Lausanne). 2025 Jan 7;5:1484948. doi: 10.3389/fpain.2024.1484948 (PMC11746909; doi:10.3389/fpain.2024.1484948)
Supplement: Supplementary file 1 [file Table1.docx]

Supplement Table 1: Propensity Matching Demographics

|  | Adult  IV ibuprofen  N=31,046  N (%) | Adult  Ketorolac  N=124,184  N (%) | Pediatric  IV ibuprofen  N=5,579  N (%) | Pediatric  Ketorolac  N=5,579  N (%) |
| --- | --- | --- | --- | --- |
| Age |  |  |  |  |
| 0-5 | - | - | 38% | 38% |
| 6-11 | - | - | 34% | 34% |
| 12-17 | - | - | 28% | 28% |
| 18-32 | 17% | 17% | - | - |
| 33-44 | 19% | 19% | - | - |
| 45-54 | 15% | 15% | - | - |
| 55-64 | 21% | 21% | - | - |
| 65+ | 27% | 27% | - | - |
| Gender |  |  |  |  |
| Male | 33% | 33% | 52% | 52% |
| Reason for drug administration |  |  |  |  |
| Surgery | 94% | 94% | 44% | 44% |
| Fever | 0.38% | 0.38% | 19% | 19% |
| Non-surgical | 5.2% | 5.2% | 36% | 36% |
